# Genomic and Phenotypic Analysis of an ESBL-Producing *E. coli* ST1159 Clonal Lineage From Wild Birds in Mongolia

**DOI:** 10.3389/fmicb.2020.01699

**Published:** 2020-07-21

**Authors:** Peter Schierack, Stefan E. Heiden, Muhammad Moman Khan, Lena Nikolaus, Rafal Kolenda, Michael Stubbe, Davaa Lkhagvasuren, Stefan Rödiger, Sebastian Guenther, Katharina Schaufler

**Affiliations:** ^1^Multiparametric Diagnostics, Brandenburg University of Technology Cottbus – Senftenberg, Senftenberg, Germany; ^2^Institute of Pharmacy, University of Greifswald, Greifswald, Germany; ^3^Institute of Biology, Martin-Luther-University Halle-Wittenberg, Halle (Saale), Germany; ^4^Department of Biology, National University of Mongolia, Ulaanbaatar, Mongolia; ^5^Faculty of Health Sciences, Joint Faculty of the Brandenburg University of Technology Cottbus – Senftenberg, The Brandenburg Medical School Theodor Fontane and the University of Potsdam, Senftenberg, Germany

**Keywords:** ESBL – *E. coli*, ST1159, Mongolia, wild birds, environmental epidemiology

## Abstract

**Background:**

In addition to the broad dissemination of pathogenic extended-spectrum beta-lactamase (ESBL)-producing *Escherichia (E.) coli* in human and veterinary medicine and the community, their occurrence in wildlife and the environment is a growing concern. Wild birds in particular often carry clinically relevant ESBL-producing *E. coli.*

**Objectives:**

We analyzed ESBL-producing and non-ESBL-producing *E. coli* obtained from wild birds in Mongolia to identify phylogenetic and functional characteristics that would explain the predominance of a particular *E. coli* clonal lineage in this area.

**Methods:**

We investigated ESBL-producing *E. coli* using whole-genome sequencing and phylogenetics to describe the population structure, resistance and virulence features and performed phenotypic experiments like biofilm formation and adhesion to epithelial cells. We compared the phenotypic characteristics to non-ESBL-producing *E. coli* from the same background (Mongolian wild birds) and genomic results to publicly available genomes.

**Results and Conclusion:**

We found ESBL-producing *E. coli* sequence type (ST) 1159 among wild birds in Mongolia. This clonal lineage carried virulence features typical for extra-intestinal pathogenic or enterotoxigenic *E. coli.* Comparative functional experiments suggested no burden of resistance in the ST1159 isolates, which is despite their carriage of ESBL-plasmids. Wild birds will likely disseminate these antibiotic-resistant pathogens further during migration.

## Introduction

Extended-spectrum beta-lactamase (ESBL)-producing *Escherichia (E.) coli* presents a major threat to public health worldwide (Ferri et al., 2017). Pathogenic representatives cause a range of severe infectious diseases in humans and animals including sepsis, urinary tract and wound infections (Ewers et al., 2012). In addition to their increasing prevalence as infectious agents in human and veterinary medicine, their occurrence in wildlife and the environment is of great concern. It has been previously shown that wild birds carry ESBL-producing *E. coli* of different sequence types (ST) (Schaufler et al., 2015, 2019; Guenther et al., 2017). However, we know little about factors that influence the spread of these pathogens in the absence of antibiotic selection pressures (Nicolas-Chanoine et al., 2013). In this study, we report the broad occurrence of an ESBL-producing *E. coli* ST1159 clonal lineage among wild birds in Mongolia, its phylogenetic and functional characteristics and phenotypic differences to other STs and non-ESBL-producing *E. coli* from the same background.

## Materials and Methods

### Origin and Isolation of Bacteria

**FIGURE 1:**
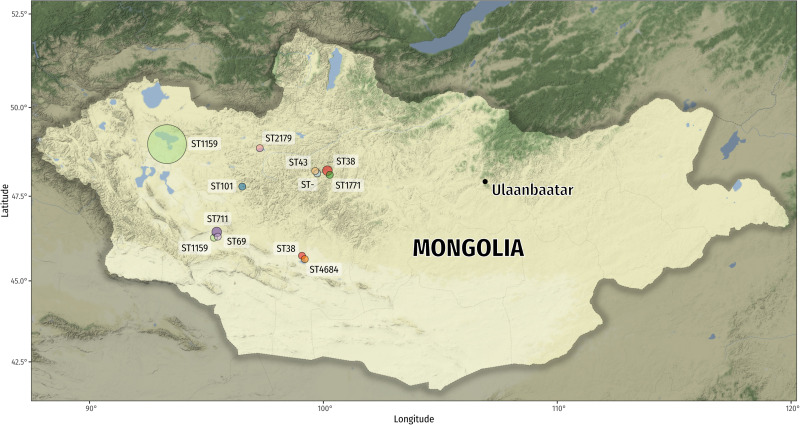
Map of Mongolia depicting the sample locations of the various *E. coli* isolates. The circle area is proportional to the number of sequence types recovered (ST1159: *n* = 36 and *n* = 1; ST38: *n* = 2 and *n* = 1; ST711: *n* = 2; all other STs: *n* = 1). The new sequence type (ST-) is a single-locus variant (SLV) of ST2179 (1 single-nucleotide variant [SNV] in *fumC*). Map tiles by Stamen Design, under CC BY 3.0. Data by OpenStreetMap, under ODbL.

Cloacal swabs of apparently healthy nestlings and juvenile wild birds were obtained in different remote areas of Mongolia during a period of 4 weeks during July 2017 as part of a “bird ringing expedition” (Figure 1). Samples of cormorants were obtained in three large bird colonies (*n* > 1000 individuals) during the ringing procedure, samples of birds of prey while ringing birds from individual nests and cranes were caught and sampled individually (without ringing). The sampling locations in this study are among the least densely human-populated areas in the world and large cities were not present (Guenther et al., 2012, 2017; Nicolas-Chanoine et al., 2013). With the exception of moderate ruminant farming, no agricultural activities (e.g., fertilizing fields with manure) were recorded around the sampling locations for hundreds of kilometers. We did not choose any areas with free-ranging livestock for sampling. Overall, 316 individual birds were included (241 cormorants [*P. carbo*], 37 cranes [*A. virgo*], and 38 other birds [*A. monachus*, *M. migrans, F. tinnunculus, A. cinerea*]). Cloacal swabs (Copan fecal swab, MAST) were shipped to Germany and streaked on agar plates to isolate coliform-appearing bacteria. Figure 1 and Supplementary Table 1 provide details on geographic sampling locations and host species of obtained bacteria. *E. coli* (both ESBL- and non-ESBL-producers) were isolated from selective chromogenic (MAST CHROMagar) plates with and without 4 μg/mL cefotaxime (the latter for ESBL-producers). One single colony per plate was selected and ESBL-producers were differentiated from AmpC-producers using the Vitek V2 System (Biomerieux, France). Overall, we obtained 49 ESBL-producing *E. coli* isolates. In addition, we analyzed 42 non-ESBL-producing *E. coli* from colony 2 (Airag Nur) to phenotypically compare the isolates.

### Bioinformatics Analysis

All ESBL-producing *E. coli* (*n* = 49; [group 1: all ST1159 isolates and group 2: all non-ST1159 ESBL-isolates]) were whole-genome sequenced on an Illumina MiSeq (2× 300 bp PE) in collaboration with Eurofins Genomics (Ebersberg, Germany). Raw reads were quality-trimmed, adapter-trimmed and contaminant-filtered using BBDuk from BBTools v. 38.41^1^. After *de novo* assembly of trimmed reads into contiguous sequences (contigs) using shovill v. 1.0.4 in combination with SPAdes v. 3.13.1^2^ (Bankevich et al., 2012), draft genomes were polished by mapping trimmed reads back to the contigs with bwa v. 0.7.17-r1188 (Li and Durbin, 2009) and calling SNPs and indels with Pilon v. 1.23 (Walker et al., 2014). Putative plasmid sequences were extracted using mlplasmids (Arredondo-Alonso et al., 2018) and compared to the NCBI database using BLASTN^3^. Then, we detected sequence types, antibiotic resistance/virulence genes and SNPs using mlst v.2.17.6, abricate v.0.9.8 (including the following databases: Resfinder, CARD, ARG-ANNOT, NCBI ARRGD, EcOH, PlasmidFinder, VFDB and Ecoli_VF) and snippy v.4.4.1^4^ ^,5^ ^,6^. We inferred a core SNP phylogeny for ST1159. For this, alignments were filtered for recombination using Gubbins v.2.3.4 (Croucher et al., 2015) and core SNPs extracted using snp-sites v.2.4.1 (1675 sites; Page et al., 2016). We inferred a maximum likelihood tree with RAxML-NG v.0.9.0 (Kozlov et al., 2019). The best-scoring maximum likelihood tree was midpoint-rooted and visualized in FigTree^7^. By applying Roary v. 3.12.0 (Page et al., 2015), we assessed the pan genome of the isolates and visualized it together with the phylogeny and metadata using Phandango (Hadfield et al., 2018). We included twelve publicly available ST1159 genome sequences^8^, which were processed as mentioned above and one provided (from colleagues at the Institute of Microbiology and Epizootics at Freie Universität Berlin) ST1159 isolate: PBIO1289 (*n* = 13; [group 3]). Visualization of *E. coli* virulence factors^9^ was done with BRIG v.0.95-dev-0004 (Blast Ring Image Generator) (Alikhan et al., 2011). The synteny visualization of ST1159 isolate PBIO845 with UPEC strain UMN026 and ETEC strain UMNK88 was created with tblastx (query and database genetic code: 11; *E*-value: 1e-10; Camacho et al., 2009) and genoPlotR v. 0.8.9 (minimum bitscore: 50; Guy et al., 2010).

### Construction of an ESBL-Plasmid-Cured Variant (PCV)

We constructed an ESBL-plasmid-free variant of one of the ST1159 isolates (PBIO845) by growing isolates at 42°C in BHI broth with daily subcultivations for 4 weeks (Schaufler et al., 2016). To confirm loss of the large ESBL-plasmid, we performed plasmid profile analysis and PCR as described previously (Schaufler et al., 2013).

### Phenotypic Experiments

We carried out phenotypic experiments for the 49 ESBL- and 42 non-ESBL-producers and PCV to investigate potential phenotypic differences between and within bacterial groups. Experiments included adhesion (Frommel et al., 2013), long-term colony (Schaufler et al., 2016), and acidity tolerance assays. To test adhesion to LoVo and CHIC-8E11 epithelial cells, we used a 96-well-plate-based assay, measured adherence with a fluorescence microscope (VideoScan system) (Rodiger et al., 2013) and calculated adhesion ratios accordingly. All cells were grown and adhesion assays were carried out as previously described (Frommel et al., 2013). *E. coli* were grown overnight to an OD_600_ of 0.8–1.2. Cells were inoculated with an infection dose of about 62,500 bacteria per mm^2^ of a monolayer using a conversion of 3×10^8^ bacteria/mL/OD_600_. After 4 h of incubation and washing with 1×PBS, cells and adherent bacteria were fixed with 4% paraformaldehyde. Cells and bacteria were stained with propidium iodide (10 μg/mL in ddH_2_O) and analyzed. A minimum cell monolayer area of 0.3 mm^2^ per well was investigated. All 150,000 images were visually analyzed for *E. coli* pattern formation. Isolates whose images resembled those of the negative control (eukaryotic cells without bacteria) were defined as non-adherent.

Then, we tested the isolates on long-term colony plates to assess their ability to form biofilm-related structures. Three microliters of overnight cultures grown at 37°C were dropped on span agar plates (H. Carroux, Germany) with sodium chloride (5%) and a 0.5% Congo red–0.25% Coomassie brilliant blue solution. Plates were incubated for 5 days at 28°C and screened for curli fibers and cellulose production. A purple long-term colony color indicates the production of curli fibers; elevated, structured surfaces indicate the production of cellulose. Plain white colonies indicate the absence of both (Bokranz et al., 2005). Acidity tolerance was tested in 96-well-plates with different pH values (highest at 7, lowest at 3.5 with 0.5 pH steps). Tolerance to the respective pH value was defined as growth of the isolate in the respective well after 24 h of incubation at 37°C.

All phenotypic experiments were performed in a minimum of three biological replicates and statistically evaluated using the Mann-Whitney U-test and Fisher’s Exact Test with Bonferroni correction to correct for the multiple comparisons and account for non-normally distributed values (*p* value < 0.0125).

## Results and Discussion

Over two thirds (37/49) of ESBL-producing *E. coli* belonged to ST1159 (group 1; phylogroup B2). Thirty-five ST1159 were obtained from one bird colony and one bird species (Great Cormorant, 46% of the sampled nestlings). The other two ST1159 isolates originated from two species (Black Kite and Gray Heron) suggesting inter-host exchange, however, it is also possible that the isolates occurred independently of each other. The Gray Heron was sampled in the same colony as the cormorants, the Black Kite sample originated from nestlings sampled 300 km away (Figure 1). Other sequence types among the ESBL-producers included ST38 (*n* = 3; phylogroup D), ST711 (*n* = 2; phylogroup B1) and others (group 2) (Supplementary Table 1). The new sequence type (ST-) is a single-locus variant (SLV) of ST2179 caused by one single-nucleotide variant (SNV) in *fumC* allele 65 (C > T; Ala > Val). Previously, we have found ST38 in Mongolian wild birds that contained chromosomally encoded ESBL-genes (Guenther et al., 2017), however, in this study bioinformatics analysis suggested plasmid-encoded *bla*-genes in ST38.

**FIGURE 2:**
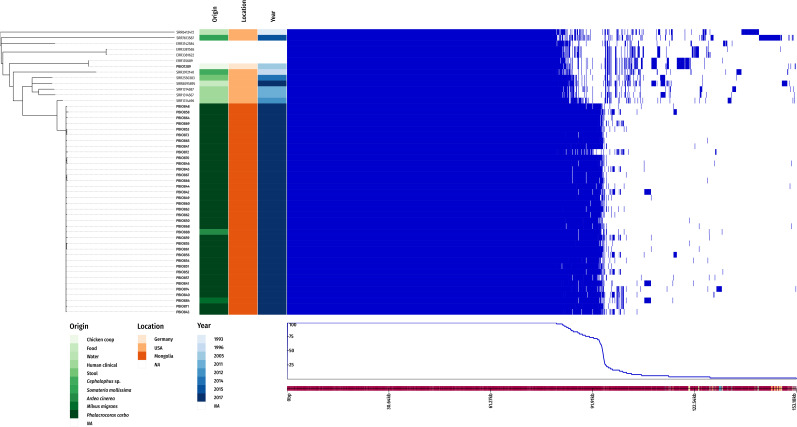
*E. coli* ST1159 core SNP phylogeny. The midpoint-rooted phylogenetic tree was inferred using a maximum likelihood approach with the GTR model of nucleotide substitution and a gamma model of rate heterogeneity. It is depicted with metadata where available and the output of the Roary pangenome analysis. Isolate names in bold designate isolates whose genomes were sequenced as part of this study. Names starting with ERR or SRR designate raw read accession numbers of publicly available ST1159 isolates.

Figure 2 reveals that all 37 ST1159 from Mongolian wild birds belong to a single clonal lineage (max. 1.4 SNPs/aligned Mbp). The twelve publicly available ST1159 genomes and PBIO1289 (group 3), the latter isolated from a duck coop in Germany in 2005 (Ewers et al., 2009) were further distant, which is not surprising given the fact that they are geographically and timely dispersed and that SNPs accumulate over time. Interestingly, the closest “external” ST1159 to group 1 isolate is a clinical extra-intestinal pathogenic *E. coli* (ExPEC) isolated from a urinary tract infection (Salipante et al., 2015). The duck sample was identified as avian pathogenic *E. coli* (APEC) (Ewers et al., 2009). It is well known that ExPEC and APEC share common pathogenic features and often belong to pathogenicity-associated phylogroup B2 (Ewers et al., 2007) which does not only underline the clinical relevance of ESBL-producing ST1159 group 1 but also serves as possible explanation for the success of ST1159 among birds.

**FIGURE 3:**
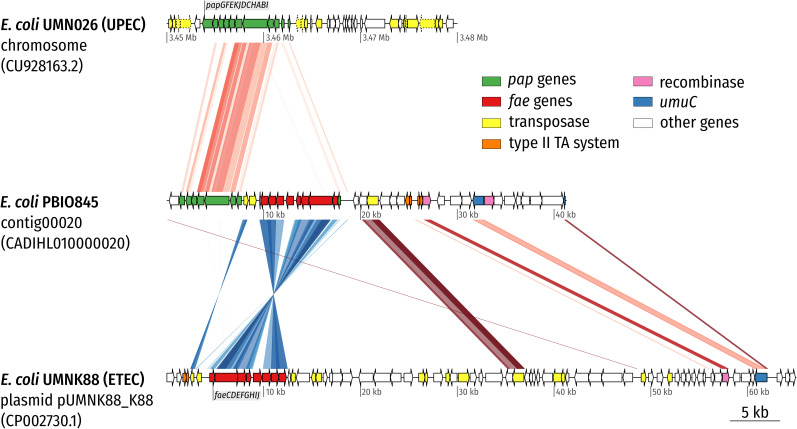
Synteny plot of *E. coli* PBIO845 (ST1159) contig00020. The plasmid-derived contig was aligned with the chromosome of UPEC strain UMN026 and plasmid pUMNK88_K88 of ETEC strain UMNK88 using tblastx (*E*-value 1e-10). The contig combines features commonly found in UPEC strains (P pili encoded by the *pap* operon) with ETEC-associated features (K88 [F4] fimbriae encoded by the *fae* operon). Direct comparisons are colored with red hues whereas reverse comparisons are colored with blue hues. Arrows depict coding sequences (CDS) and are colored according to the legend. Arrows with a dotted outline are annotated as pseudo CDS. While the complete sequence of contig00020 is shown (middle), the chromosome (top) and plasmid sequences (bottom) were truncated in the displayed region.

Bioinformatics and plasmid profile analysis suggested that group 1 ST1159 isolates shared identical plasmid contents, namely two large plasmids (∼90–125 kb each), of which one encoded the ESBL-enzyme (CTX-M-14) and the other several virulence features. Determined plasmid incompatibility (Inc.) types were IncFIC(FII) and IncI1. Both IncF- as well as IncI-type plasmids have been previously associated with the spread of ESBL-producing *E. coli* (Coque et al., 2008; Branger et al., 2018; Montealegre et al., 2020). Group 3 genomes showed only small plasmid analogies with the Mongolian representatives and did not carry any ESBL-genes. Interestingly, the group 1 plasmid sequences combined chromosomally encoded features of uro-pathogenic *E. coli* (which belong to ExPEC) – the *pap*-like operon (P pili, PAI [pathogenicity island] I(APEC-O1)) – as well as *fae* genes (PAI: K88 [F4^+^] fimbriae), which have previously been described on plasmids from enterotoxigenic *E. coli* (Figure 3; Shepard et al., 2012).

We were surprised by the high number of ST1159 isolates and thus focused on factors that would explain their broad occurrence. First, we investigated the carriage of virulence genes among ST1159 and non-ST1159 genomes. We identified a set of clinically relevant virulence factors (Ewers et al., 2007) i.e., *fae* (fimbrial proteins), *vat* (vacuolating autotransporter toxin), *malX* (maltose transporter), *usp* (uropathogenic specific protein), and *ibeA* (invasion protein), which were unique to group 1 and absent to group 2. Some of these were present in the external ST1159 group 3 (Supplementary Figure 1). For example, all external genomes carried *vat, malX* and *ibeA*. Note, however, that malX, a phosphotransferase system enzyme, is present in most isolates of *E. coli* and may be overestimated regarding its importance as virulence factor. Other virulence features were present in all groups (Supplementary Figure 1). We then investigated the potential role of several of these virulence features in functional experiments. As *fae* genes have been previously described as epithelial adhesins (Xia et al., 2015) we compared groups 1 and 2 as well as non-ESBL-producing isolates (*n* = 42; [group 4]) in adhesion experiments. We observed no significant differences in bacterial adherence to gut epithelial chicken cells (CHIC) or to those of human origin (LoVo cells). Next, we investigated phenotypes potentially relevant to the success of bacteria in wild birds and the environment. Biofilm formation is an important virulence feature, and associates to increased environmental survival (Schaufler et al., 2019). Interestingly, all group 1 isolates except one formed biofilm-associated curli and cellulose components, which was significantly better than group 4 (*p* = 0.003). This might point toward an advantage in tolerating environmental challenges when disseminating within and across bird colonies. It is known that bird droppings contain uric acid resulting in low pH values. We performed dilution series to investigate whether ST1159 was more tolerant to acidity than others. The non-ESBL-producers (group 4) showed no significantly better growth in low pH values than ST1159 (group 1). In an earlier study, we report that ESBL-plasmids increased virulence in some *E. coli* strains. We thus constructed a ST1159 (PBIO845) ESBL-plasmid-cured variant and included it in the above-mentioned phenotypic experiments. We did not observe any virulence benefit in the respective ST1159 wildtype when compared to the PBIO845 mutant.

Our results do not clarify unequivocally why the ESBL-producing ST1159 clonal lineage proliferated in wild birds from Mongolia. ST1159 is a rare sequence type, previously described a dozen times only. Given that we found this ST in 76% of all obtained ESBL-producing isolates from Mongolian wild birds suggests, however, that it has developed unique strategies to adapt to this ecology rather than arising from pure evolutionary coincidence. The intra-host diversity of *E. coli* is generally high. In wildlife even more than in domestic animals (Escobar-Paramo et al., 2006; Tenaillon et al., 2010). The occurrence of a single ESBL-producing *E. coli* clonal lineage and its spread within one bird colony and to other individuals is thus surprising, especially when taking the low anthropogenic impact in this area into account. The population density of Mongolia is about two people per square kilometer (for comparison: EU ∼ 250/km^2^, India ∼ 450/km^2^, and China ∼ 150/km^2^), and even lower at the Airag Nur (1/km^2^). Within 50 kilometers of this lake, only one small permanent settlement is present (Zavkhan ∼ 50–100 inhabitants), and the next village with a medical service is 80 kilometers away (Urgamal ∼1200 inhabitants). Uliastai, which is a city with about 10.000 inhabitants is located 400 kilometers upstream of the river Zavkhan. In addition, as intensive livestock and agricultural farming was completely absent in our sampling locations, the presence of antibiotic residues seems highly unlikely. The available knowledge indicates that different factors are responsible for the success of *E. coli* clones. In all experiments, ST1159 isolates –despite carrying ESBL-plasmids- were not inferior to the non-ESBL-producing group and showed significantly more often a production of curli and cellulose. These biofilm-associated components might contribute to the environmental success and spread of ST1159 in bird populations. ST1159 proliferated in these wild birds, which suggests that the frequent detection of ESBL-producing isolates in wildlife is not just based on spill-over effects but the expansion of certain clonal lineages able to cope with environmental challenges while simultaneously carrying resistance plasmids.

## Conclusion

We speculate that an adult bird has introduced ST1159 to the cormorant colony and/or the Airag Nur lake first. Then, it might have spread among the cormorant nestlings, nearby Heron nestlings at the same lake, and long distance to a 300 km-away bird of prey colony (Figure 1). Cormorant colonies are densely colonized and covered in bird droppings, which results in a situation comparable to industrial poultry production. ST1159’s spread took likely place among nestlings within the colony rather than being driven by repeated introductions through contaminated fish from the lake. Fish are not a natural host for *E. coli* bacteria and fecal pollution of the lake is unlikely. As cormorants are migrating birds using the Central Asian flyway (Prosser et al., 2011) the origin of the ST1159 clonal lineage might be somewhere in the avian wintering grounds in the southern parts of Central Asia, where anthropogenic impacts are high. Nestlings hatching in this “bio-fermenters-like colony” may further spread ST1159 during their winter migration southwards, similar to what has been shown for cormorants carrying the H5N1 influenza virus (Hu et al., 2011). Keep in mind, however, that the biased sample set with a majority of ESBL-producing isolates and ST1159 obtained from mostly one single cormorant colony does not allow to suggest that this is a generally valid finding for the different locations in Mongolia.

## Data Availability Statement

The datasets generated for this study can be found in the European Nucleotide Archive (ENA) at EMBL-EBI with the accession number PRJEB36861 (https://www.ebi.ac.uk/ena/data/view/PRJEB36861).

## Author Contributions

PS, KS, MS, and SG conceived and designed the experiments. PS, SG, MS, and DL collected the data and samples. PS, KS, MK, LN, RK, and SR performed laboratory analysis. KS, SG, PS, and SH analyzed the data and wrote the manuscript. All authors have read and approved the final draft of the manuscript.

## Conflict of Interest

The authors declare that the research was conducted in the absence of any commercial or financial relationships that could be construed as a potential conflict of interest.

## Footnotes

1 http://sourceforge.net/projects/bbmap

2 https://github.com/tseemann/shovill

3 https://blast.ncbi.nlm.nih.gov/Blast.cgi

4 https://github.com/tseemann/mlst

5 https://github.com/tseemann/abricate

6 https://github.com/tseemann/snippy

7 https://github.com/rambaut/figtree

8 https://enterobase.warwick.ac.uk/

9 https://github.com/phac-nml/ecoli_vf

## Supplementary Material

FIGURE S1 BLASTN comparison (*E*-value 1e-10, low complexity filter turned off) of *E. coli* genomes versus selected *E. coli* virulence factors. Each concentric ring (from inner to outer) belongs to an isolate as depicted in the legend (top to bottom). Group 1 (ST1159) and 2 (Non-ST1159) contain isolates recovered from Mongolian samples. Group 3 consists of publicly available ST1159 isolates (raw read accession number given) and one isolate originating from a duck coop in 2005.

TABLE S1 Overview for ESBL-producing *E. coli:* sampled avian host species, sample types, sampling location coordinates, sampling dates, identified *E. coli* multi-locus sequence types (STs), resistance genes and plasmid incompatibility groups based on the whole-genome sequences using mlst, abricate, and snippy.
